# Towards detection of early response in neoadjuvant chemotherapy of breast cancer using Bayesian intravoxel incoherent motion

**DOI:** 10.3389/fonc.2023.1277556

**Published:** 2023-12-06

**Authors:** Sai Man Cheung, Wing-Shan Wu, Nicholas Senn, Ravi Sharma, Trevor McGoldrick, Tanja Gagliardi, Ehab Husain, Yazan Masannat, Jiabao He

**Affiliations:** ^1^ Institute of Medical Sciences, School of Medicine, University of Aberdeen, Aberdeen, United Kingdom; ^2^ Department of Oncology, Aberdeen Royal Infirmary, Aberdeen, United Kingdom; ^3^ Department of Radiology, Royal Marsden Hospital, London, United Kingdom; ^4^ Department of Pathology, Aberdeen Royal Infirmary, Aberdeen, United Kingdom; ^5^ Breast Unit, Aberdeen Royal Infirmary, Aberdeen, United Kingdom; ^6^ Translational and Clinical Research Institute, Faculty of Medical Sciences, Newcastle University, Newcastle upon Tyne, United Kingdom

**Keywords:** diffusion, cellularity, microcirculation, perfusion fraction, pathological response

## Abstract

**Introduction:**

The early identification of good responders to neoadjuvant chemotherapy (NACT) holds a significant potential in the optimal treatment of breast cancer. A recent Bayesian approach has been postulated to improve the accuracy of the intravoxel incoherent motion (IVIM) model for clinical translation. This study examined the prediction and early sensitivity of Bayesian IVIM to NACT response.

**Materials and methods:**

Seventeen female patients with breast cancer were scanned at baseline and 16 patients were scanned after Cycle 1. Tissue diffusion and perfusion from Bayesian IVIM were calculated at baseline with percentage change at Cycle 1 computed with reference to baseline. Cellular proliferative activity marker Ki-67 was obtained semi-quantitatively with percentage change at excision computed with reference to core biopsy.

**Results:**

The perfusion fraction showed a significant difference (*p* = 0.042) in percentage change between responder groups at Cycle 1, with a decrease in good responders [−7.98% (−19.47–1.73), *n* = 7] and an increase in poor responders [10.04% (5.09–28.93), *n* = 9]. There was a significant correlation between percentage change in perfusion fraction and percentage change in Ki-67 (*p* = 0.042). Tissue diffusion and pseudodiffusion showed no significant difference in percentage change between groups at Cycle 1, nor was there a significant correlation against percentage change in Ki-67. Perfusion fraction, tissue diffusion, and pseudodiffusion showed no significant difference between groups at baseline, nor was there a significant correlation against Ki-67 from core biopsy.

**Conclusion:**

The alteration in tumour perfusion fraction from the Bayesian IVIM model, in association with cellular proliferation, showed early sensitivity to good responders in NACT.

**Clinical trial registration:**

https://clinicaltrials.gov/ct2/show/NCT03501394, identifier NCT03501394.

## 1 Introduction

Neoadjuvant chemotherapy (NACT) is increasingly used in breast cancer, evolving from originally downstaging inoperable breast tumours to allow surgical excision (1) to facilitating potential breast and axillae conservation (2). However, NACT not only is costly at an estimated £6,000 per patient for a typical six-cycle regimen of 5-fluorouracil/epirubicin/cyclophosphamide (FEC 100) in the National Health Service (3) but also often leads to adverse side effects and subsequent severe physical and emotional distress (4, 5). Although NACT improves rates of pathological complete response (pCR) (6, 7) and disease-free survival (7, 8), poor responders to NACT might receive earlier and timely mastectomy or breast conservation (9). RECIST criterion, the current approach to estimate residual disease load based on tumour size (10) at the halfway point of NACT (11), has limited accuracy at a relatively late stage of treatment, demanding more precise radiological approaches.

The loss of tumour cellularity is the central histological marker of cellular damage in tumours responding to NACT (12). Diffusion-weighted imaging (DWI), although sensitive to cellularity (13, 14) with the potential of identifying responders after one cycle of NACT (15), is susceptible to biological noise and limited to large cohort studies (16), and is therefore inadequate for response-guided NACT (17). Apparent diffusion coefficient (ADC) from DWI (18) is effective in differentiation of tumour from healthy tissue and benign lesions (19, 20). An increase in ADC at the halfway point of 12 weeks of NACT-predicted pCR, however, may not reach clinical relevance with the receiver operating characteristics curve at an area under 0.6 (21). Diffusion tensor imaging yielded a significant increase in prime diffusion coefficient (*λ*_1_) and ADC in good responders compared to poor responders at the completion of NACT, although baseline diffusion metrics did not predict good response (22). Diffusion kurtosis imaging approximates the deviation from the tensor model using kurtosis, with a lower mean kurtosis at baseline associated with pCR at four cycles of NACT in patients with breast cancer (23). We have shown that *q*-space imaging was more effective in the evaluation of cellularity in breast cancer; however, the method was not suitable for routine clinical application due to the demand on high field gradient and long scan duration (24). Intravoxel incoherent motion (IVIM), incorporating tissue diffusion and blood microcirculation as two independent components (18), showed improved diagnostic sensitivity in breast cancer (25). However, IVIM is prone to misfitting as a result of the high susceptibility in the main algorithm to biological noise (26). A Bayesian probability (BP) approach has been suggested to improve fitting accuracy and reduce variability in the estimation of tissue diffusion and blood microcirculation (27).

We therefore hypothesise that the Bayesian IVIM model may differentiate good from poor responders at baseline and after Cycle 1 of NACT with association from tumour proliferative activity, providing a non-invasive biomarker sensitive to prediction and early response to NACT.

## 2 Materials and methods

**Figure 1:**
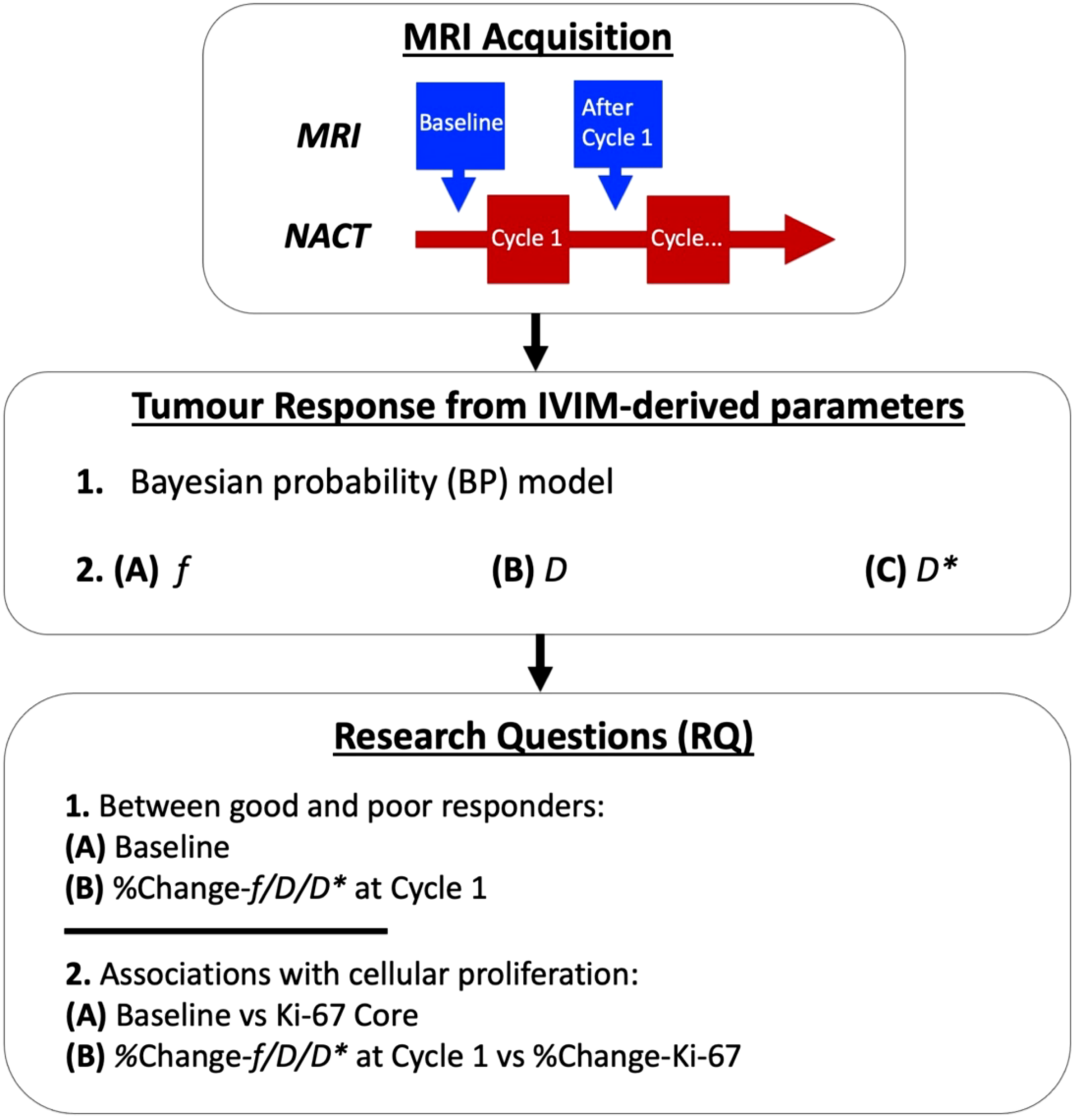
Intravoxel incoherent motion (IVIM) images were acquired before neoadjuvant chemotherapy (NACT) at baseline and after Cycle 1. A Bayesian probability (BP) IVIM model was used to compute perfusion fraction (*f*), tissue diffusion (*D*), and pseudodiffusion (D*) for the assessment of prediction and early tumour response to NACT. The baseline and percentage change in *f*, *D*, and D* at Cycle 1 were examined between good and poor responders, with patients grouped according to the Miller–Payne system for pathological response (RQ1). Medians of baseline and percentage change in *f*, *D*, and D* were compared against tumour cellular proliferation marker Ki-67 at core biopsy and percentage change in Ki-67, respectively, from immunostaining in histopathology (RQ2).

We hence conducted a prospective, longitudinal study of NACT in 17 female patients with breast cancer using the Bayesian IVIM model (**Figure 1**). The study was approved by the London Research Ethics Committee (Identifier: 17/LO/1777) and registered as a clinical trial [NCT03501394]. The planned study incorporated four MRI scans across the entire NACT, but was interrupted and closed prematurely due to the COVID-19 pandemic. Therefore, analysis was conducted on MRI scans acquired at baseline and after Cycle 1 only.

*Clinical Procedure:* Seventeen female patients (age 37–71 years), with grade II or III invasive breast carcinoma from core biopsy and planned for NACT were recruited into the study. Patients with a previous history of breast cancer or receiving hormonal treatment were not eligible. All patients received 5-fluorouracil 500 mg/m^2^, epirubicin 100 mg/m^2^, and cyclophosphamide 500 mg/m^2^ (FEC) once every 21 days for the first three cycles, and docetaxel 100 mg/m^2^ once every 21 days for the remaining three cycles (28, 29). Two patients with HER2-positive breast cancer additionally received pertuzumab and trastuzumab for a year (30, 31). MRI scans were performed at 5–10 days (median: 7) before the start of the treatment and 10–14 days (median: 12) after Cycle 1. MRI was acquired from 17 patients at baseline and 16 patients at Cycle 1 due to complications in one patient. Standard clinical histopathological examination was performed for each patient to determine histological grade, and immunostaining of Ki-67, a nuclear marker of cellular proliferation associated with worse survival outcomes (32), was conducted in a single batch. The histology results were obtained from core biopsies before NACT and resected residual tumours after six cycles respectively, with appropriate positive controls (33). The pathological response was assessed on the resected tumours, and the good responders and poor responders were identified as above (grades 4 and 5) or below (grades 1, 2, and 3) 90% reduction in cellularity, respectively, according to the Miller–Payne system (12). The percentage change in Ki-67 was computed as the difference between biopsy and excision, normalised to biopsy: [Ki-67 in resected tumour – Ki-67 in core biopsy]/Ki-67 in core biopsy × 100%.

*Magnetic Resonance Imaging:* All images were acquired on a 3 T clinical whole-body MRI scanner (Achieva TX, Philips Healthcare, Best, The Netherlands), using body coil for uniform transmission and a 16-channel breast coil for signal detection. Patients were in prone position with the imaging volume centered on the breast affected by tumour. IVIM images were acquired in the sagittal orientation using pulsed gradient spin echo (PGSE) sequence with single-shot echo planar imaging (EPI) at 10 diffusion weightings (*b*-values at 0, 30, 60, 90, 120, 250, 400, 600, 800, and 1,000 s/mm^2^) (34). For each *b*-value, diffusion gradients were applied along three orthogonal directions, and the image was computed as the average across the three directions. Images were acquired with a diffusion time (δ/Δ) of 13.1/25.4 ms, a field of view (FOV) of 240 mm × 240 mm, an in-plane resolution of 2.5 mm × 2.5 mm, a slice thickness of 5 mm, an acceleration factor of 2, a repetition time (TR) of 2,400 ms, and an echo time (TE) of 50 ms.

*Image Analysis:* Bayesian IVIM was performed in MATLAB (R2020a, Mathworks, Natick, MA, USA). The tumour was delineated on dynamic contrast-enhanced MRI by a consultant radiologist in ImageJ (v1.58k, National Institute of Health, Bethesda, MD, USA), with adjustment of image resolution to match IVIM images and conservative definition of tumour boundary to avoid the necrotic, hemorrhagic, and cystic areas. The size of the tumours was evaluated based on the longest diameter from the high-resolution dynamic contrast-enhanced (DCE)-MRI (21, 35, 36). The Bayesian algorithm estimated the joint posterior distribution using the Rician noise likelihood function and uniform joint prior distribution, based on previous literature for Bayesian IVIM model fitting (37). The Bayesian fitting used a Markov chain Monte Carlo setup with Gibbs sampling and Metropolis-Hastings algorithm to derive a marginalised parameter distribution. The step-length parameters were updated every 2,000 iterations, with a total of 20,000 iterations. The conventional IVIM analysis algorithms, including nonlinear least squares full fitting, segmented-unconstrained, and segmented-constrained (38), were also deployed in supplementation to the study (**Supplementary Data: Appendix A**). The correction for the noise floor (39) was not undertaken since the data have a sufficiently high signal-to-noise ratio (SNR), and the same consistent approach was adopted for all the longitudinal data. The median perfusion fraction (*f*), tissue diffusion (*D*), and pseudodiffusion (D*) within the tumour, representing volume fraction between capillary blood and tissue water, mean diffusivity of the tissue, and vascular blood flow motion, respectively, were calculated for baseline and Cycle 1. The percentage change in perfusion fraction, diffusion, and pseudodiffusion at Cycle 1 was computed with reference to baseline: [Cycle1(*f*/*D*/D*) – Baseline(*f*/*D*/D*)]/Baseline(*f*/*D*/D*) × 100% (34).

*Statistical Analysis:* Statistical analysis was performed using the *R* software (v3.6.3, The *R* Foundation for Statistical Computing, Vienna, Austria). The normality of the distribution was assessed using the Shapiro–Wilk test. The measures at baseline and percentage change at Cycle 1 of perfusion fraction, diffusion, and pseudodiffusion were compared between good and poor responder groups using Wilcoxon rank sum test to determine the prediction and early sensitivity of the markers. The correlation of perfusion fraction, diffusion, and pseudodiffusion at baseline against Ki-67 from core biopsy for treatment-naïve prognosis was performed using Spearman’s rank correlation test. The percentage change in perfusion fraction, diffusion, and pseudodiffusion against percentage change in Ki-67 for treatment-altered prognosis was also performed using Spearman’s test. A *p*-value < 0.05 was considered statistically significant.

## 3 Results

The patient demographics is shown in **Table 1**. Among the 17 patients, there were 8 good responders and 9 poor responders at baseline, and due to complications, 1 patient did not complete an MR scan at Cycle 1. There was no significant difference in age and tumour size at baseline between good and poor responders. There was no significant difference in the change in tumour size between good and poor responders at Cycle 1 (**Table 1**).

**Table 1:** Tumour characteristics of patients.

Characteristic	All (*n* = 17)	Good Responders (*n* = 8)	Poor Responders (*n* = 9)	*p*-value
Age	51 (46–58)	50 (38–59)	52 (47–58)	NS
Tumour size at baseline (mm)	32 (26–38)	38 (34–43)	29 (20–37)	NS
Tumour size changes at Cycle 1 (%)	−7.3 (−16.7 to 0.0)	−16.7 (−27.1 to −4.4)	−3.9 (−8.3 to 0.0)	NS
*Histology*				
Invasive ductal carcinoma	16	7	9	
Mixed ductal/lobular carcinoma	1	1	0	
*Grade*				
Grade II	1	1	0	
Grade III	16	7	9	
*Hormonal receptor status*				
Oestrogen receptor positive (ER+)	7	3	4	
Human epidermal growth factor receptor 2 positive (HER2+)	2	2	0	
Triple negative (TN)	8	3	5	

Tumour histology and hormonal receptor status grouped by the Miller–Payne system (Poor Responders: 1, 2, and 3; Good Responders: 4 and 5). Median (interquartile range, IQR) of age, tumour size, and size changes are shown.

NS, not significant.

There was no significant difference in Bayesian perfusion fraction (*p* = 0.481), tissue diffusion (*p* = 0.743), and pseudodiffusion (*p* = 0.673) at baseline between good and poor responders (**Table 2**; **Supplementary Figure A1**). There was also no significant difference in perfusion fraction, tissue diffusion, and pseudodiffusion at baseline between good and poor responders from full fitting and segmented analyses (**Supplementary Table A1**, **Supplementary Figure A2**). There was no significant correlation in Bayesian perfusion fraction (*p* = 0.480), tissue diffusion (*p* = 0.174), and pseudodiffusion (*p* = 0.474) at baseline against Ki-67 from core biopsy (**Table 2**, **Supplementary Figure A1**). There was also no significant correlation in perfusion fraction, tissue diffusion, and pseudodiffusion at baseline against Ki-67 from core biopsy from full fitting and segmented analyses (**Supplementary Table A1**, **Supplementary Figure A3**).

**Table 2:** Comparison of IVIM-derived parameters between responder groups before and after the first cycle of NACT and the association with Ki-67.

IVIM-derived parameters	Baseline-f/D/D*			%Change-f/D/D*			Ki-67 correlations (*ρ* score, *p*-value)	
	All (*n* = 17)	Good Responder (*n* = 8)	Poor Responder (*n* = 9)	All (*n* = 16)	Good Responder (*n* = 7)^a^	Poor Responder (*n* = 9)	Core^b^	%Change^c^
***f^d^***	10.81 (8.89–11.71)	9.95 (8.13–11.73)	11.01 (9.34–11.71)	5.54 (−10.34 to 15.37)	−7.98 (−19.47 to 1.73)*	10.04 (5.09–28.93)*	0.180, 0.480	0.590, 0.042*
***D***	0.95 (0.88–1.27)	0.95 (0.84–1.51)	0.96 (0.91–1.19)	16.06 (2.61–32.31)	28.87 (6.98–33.77)	15.54 (0.79–25.42)	−0.350, 0.174	0.380, 0.217
D*	6.20 (4.38–9.04)	4.63 (4.03–12.83)	6.23 (6.17–8.41)	−15.50 (−19.05 to −2.14)	−16.12 (−17.05 to −3.50)	−14.89 (−24.98 to −4.12)	0.190, 0.474	0.530, 0.075

a One patient did not complete MR scan due to complications.

b Spearman’s rank correlation test – baseline-f/D/D* vs. Ki-67 Core.

c Spearman’s rank correlation test – %Change-f/D/D* vs. %Change-Ki-67.

d Units at baseline – f: percentage (%), D and D*: ×10^−3^ mm^2^/s.

The baseline and percentage change in perfusion fraction (f), tissue diffusion (D), and pseudodiffusion (D*) in good responders and poor responders from the Bayesian probability (BP) IVIM model. The Spearman’s rank correlation coefficients (ρ) for baseline IVIM-derived parameters against Ki-67 in core biopsy and percentage changes in IVIM-derived parameters against percentage change in Ki-67 are also shown. Values are presented as median (IQR). Statistically significant differences (p < 0.05) are marked with an asterisk (*).

**Figure 2:**
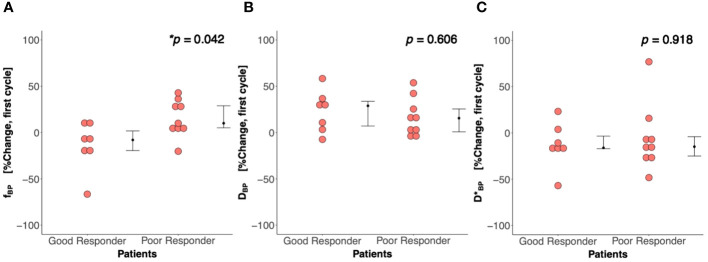
Percentage change in **(A)** perfusion fraction (*f*), **(B)** tissue diffusion (*D*), and **(C)** pseudodiffusion (D*) between good and poor responders at first treatment cycle (Cycle 1) from the Bayesian probability (BP) IVIM model. There was a significant difference in percentage change in perfusion fraction between good and poor responders, but not in tissue diffusion and pseudodiffusion. Each dot represents the percentage change in *f*, *D*, and D* from an individual patient. Error bar represents median (IQR). Statistically significant *p*-values (<0.05) are shown on the upper right corner with “*”.

**Figure 3:**
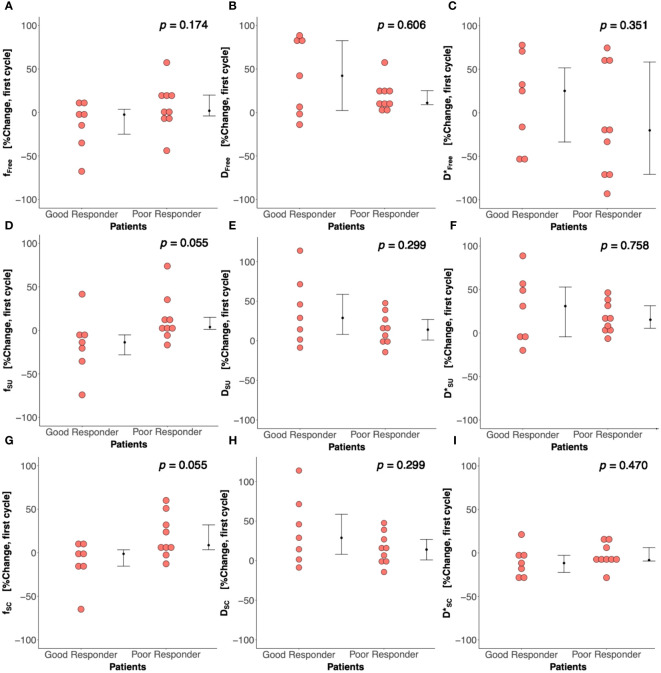
Percentage change in perfusion fraction (*f*), tissue diffusion (*D*), and pseudodiffusion (D*) between good and poor responders from nonlinear least squares (Free), segmented-unconstrained (SU), and segmented-constrained (SC) IVIM models. The percentage change in *f*, *D*, and D* between good and poor responders from **(A–C)** Free, **(D–F)** SU, and **(G–I)** SC algorithms are shown in dot plots. Each dot represents the IVIM-derived parameter of an individual patient. Error bar represents median (IQR).

**Figure 4:**
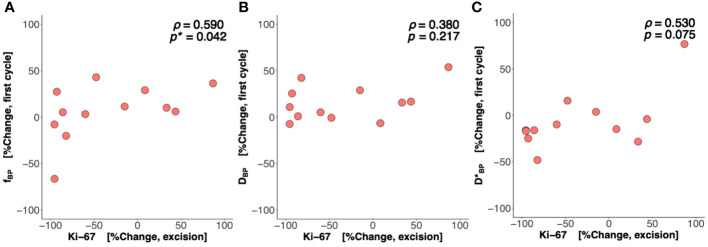
Percentage change in perfusion fraction (*f*), tissue diffusion (*D*), and pseudodiffusion (D*) against percentage change in the tumour cellular proliferation marker Ki-67. The correlation of percentage change in **(A)** *f*, **(B)** *D*, and **(C)** D* at Cycle 1 against percentage change in Ki-67 in resected tumour is shown in scatter plots. Spearman’s rank correlation coefficient (*rho* (*ρ*)) was used for correlation analysis and respective *ρ* score and *p*-value are shown on each plot. Statistically significant *p*-values (<0.05) are marked by “*”.

**Figure 5:**
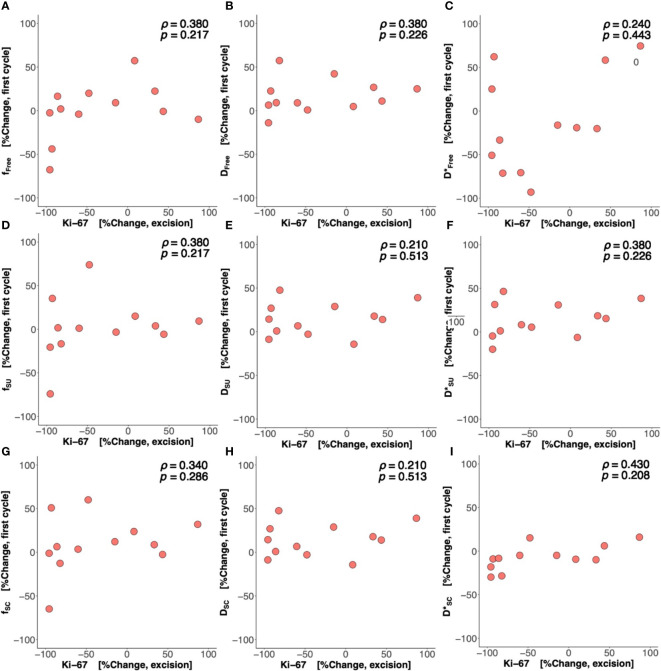
Percentage change in perfusion fraction (*f*), tissue diffusion (*D*), and pseudodiffusion (D*) against percentage change in Ki-67. The correlations of percentage change in *f*, *D*, and D* from **(A–C)** nonlinear least squares (Free), **(D–F)** segmented-unconstrained (SU), and **(G–I)** segmented-constrained (SC) algorithms at Cycle 1 against percentage change in tumour cellular proliferation marker Ki-67 in resected tumour are shown in scatter plots. Spearman’s rank correlation coefficient (*rho* (*ρ*)) was used for correlation analysis and respective *ρ* score and *p*-value are shown on each plot.

There was a significant difference (*p* = 0.042) in percentage change in Bayesian perfusion fraction between good and poor responders at Cycle 1, with a decrease in good responders [−7.98% (−19.47–1.73), *n* = 7] against an increase in poor responders [10.04% (5.09–28.93), *n* = 9] (**Figure 2A**, **Table 2**). There was no significant difference in percentage change in perfusion fraction between good and poor responders at Cycle 1 from full fitting and segmented analyses (**Figure 3**; **Supplementary Table A1**). There was a significant correlation in percentage change in Bayesian perfusion fraction (*p* = 0.042, **Figure 4A**, **Table 2**) against percentage change in Ki-67. There was no significant correlation in percentage change in perfusion fraction against percentage change in Ki-67 from full fitting and segmented analyses (**Figure 5**; **Supplementary Table A1**). There was no significant difference in percentage change in Bayesian tissue diffusion (*p* = 0.606, **Figure 2B**, **Table 2**) and pseudodiffusion (*p* = 0.918, **Figure 2C**, **Table 2**) between good and poor responders at Cycle 1. There was no significant correlation in percentage change in Bayesian tissue diffusion (*p* = 0.217, **Figure 4B**, **Table 2**) and pseudodiffusion (*p* = 0.075, **Figure 4C**, **Table 2**) against percentage change in Ki-67. There was also no significant difference in percentage change in tissue diffusion and pseudodiffusion between good and poor responders (**Figure 3**, **Supplementary Table A1**), nor was there correlation against percentage change in Ki-67 from full fitting and segmented analyses (**Figure 5**; **Supplementary Table A1**).

**Figure 6:**
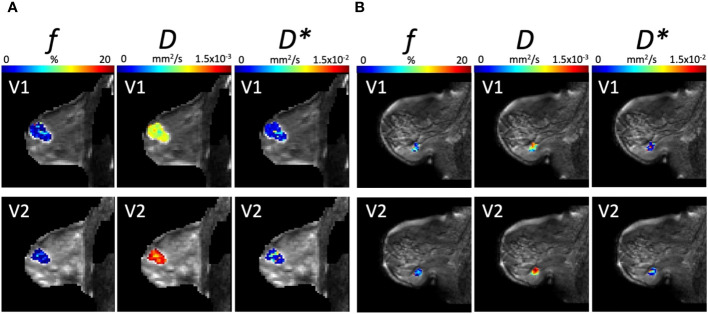
Parametric maps from IVIM Bayesian analysis of *f*, *D*, and D* from a typical **(A)** good responder and **(B)** poor responder at baseline (V1) and Cycle 1 (V2) of NACT (overlaid on diffusion weighted images, *b* = 1000 s/mm^2^). Images were acquired with a field of view of 240 mm × 240 mm, an in-plane resolution of 2.5 mm × 2.5 mm, a repetition time of 2,400 ms, and an echo time of 50 ms.

**Figure 7:**
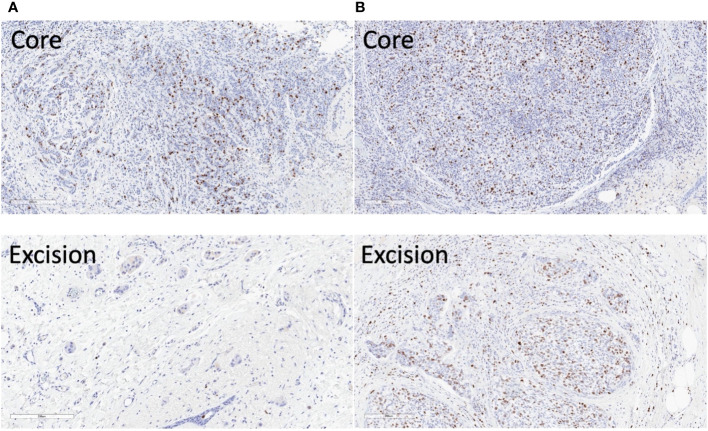
Ki-67 stained microscopy slides from a typical good and poor responder of neoadjuvant chemotherapy (NACT). **(A)** In the good responder, the Ki-67 score was 17.5% in the core biopsy and 0.8% in the resected tumour. **(B)** In the poor responder, the Ki-67 score was 23.7% in the core biopsy and 12.4% in the resected tumour. Sections at the greatest dimension of the specimens are shown. Magnification, ×10.

The parametric maps from IVIM analysis from a typical good and poor responder at baseline and Cycle 1 are shown in **Figure 6**. The Ki-67-stained microscopy slides from a typical good and poor responder at core biopsy and excision are shown in **Figure 7**.

## 4 Discussion

In this study, we investigated predictive and early response markers for NACT in breast cancer using perfusion fraction, diffusion, and pseudodiffusion derived from BP IVIM. We found that perfusion fraction showed a significant alteration between baseline and Cycle 1 in good responders compared to poor responders, and the alteration is correlated with the change in proliferative activity accumulated through the whole course of NACT across the cohort. However, we did not observe significant differences in alterations in diffusion or pseudodiffusion at Cycle 1 between groups or their correlation against change in proliferative activities. We further did not observe significant differences in imaging markers at baseline between groups, or any significant correlation against proliferative activities at baseline.

The imaging markers of tissue diffusion, perfusion fraction, and pseudodiffusion at baseline did not predict NACT response, indicating the absence of evidence to use tissue diffusion and perfusion at baseline to guide NACT. The results were in agreement with imaging markers of diffusion tensor imaging and ADC at baseline that did not have predictive value for pCR after eight cycles of NACT (22). The results also agreed with a recent study showing that pretreatment tissue diffusion, perfusion fraction, and pseudodiffusion from the segmented constrained model were not predictors of response in patients undergoing a comparable regimen of NACT (36). Diffusion and perfusion metrics estimate cellularity and angiogenesis, respectively, and the lack of a difference between responder groups indicated that a tumour with high cell density and vascular abnormality at initial presentation might not determine the effectiveness of NACT, despite an initial poorer prognosis. Diffusion and perfusion metrics showed no correlation with Ki-67 prior to NACT, indicating no direct correlation between imaging markers of cellularity and angiogenesis with treatment-naïve prognosis, although tissue sampling error could not be excluded.

There was an early significant decrease in perfusion fraction *f* in good responders, indicating that perfusion fraction might be a sensitive marker in the early identification of a successful NACT. The increase in stiffness of capillary vasculature obstructs microcirculation (40), leading to a modulation of perfusion in the tumour (41). Perfusion fraction has been shown to drastically decrease following a reduction in vascular blood flow motion, despite a subtle structural change in the functional capillary network (18). The susceptibility to systemic changes was lower in comparison to diffusion and pseudodiffusion as independent measures for the physiological response in cellularity and angiogenesis subsequent to cell apoptosis (42). Bayesian-derived perfusion fraction not only showed the potential of perfusion fraction as a marker to predict pathological complete response after one cycle of NACT, in agreement with a previous study (36), but also demonstrated a higher sensitivity since the full fitting and segmented analyses conducted in supplementation to Bayesian showed no group difference. The use of probability constraints on neighboring voxels in the Bayesian model led to less susceptibility of perfusion fraction and pseudodiffusion to the impact of noise, and improved the robustness of fitting (27). However, the higher demand on computing power may delay early adoption, whereas segmented analysis has an added advantage due to the faster processing time and initial validity in a recent study (36). Although perfusion volume ratio from DCE-MRI has been suggested as a marker of responders after one treatment cycle (43), DCE-MRI suffers from nonspecific contrast enhancement from post-treatment changes, including reactive inflammation, necrosis, and peritumoural oedema (18), requiring inputs from more than one radiologist (35). The sensitivity of DCE-MRI to angiogenesis (44) is also limited by the accuracy in the measurement of arterial input function in kinetic hemodynamic models (45) and specialist quantitative deconvolution analysis (16). The inconsistency in terminology between radiology and research practice Dickie et al. 2023^1^ further hinders the wider clinical adoption of quantitative perfusion maps from DCE-MRI for early response in NACT. IVIM, incorporating tissue diffusion and perfusion, shows clinical relevance in the current and previous studies (34, 36, 41, 46), does not require contrast, and has a clearer set of terminology to aid clinical translation. However, the higher susceptibility to noise demands extended acquisition time to reach submillimeter resolution sufficient for accurate determination of tumour size.

The significant correlation between percentage change in perfusion fraction after one cycle and percentage change in Ki-67 indicates a strong association between capillary blood-to-tumour water volume ratio with proliferative activities. Although a causal relationship for the primary impact of NACT on proliferative activity or blood supply could not be established, a reduction in metabolic demand from stunned proliferation and limitation of blood supply from restricted perfusion are both central characteristics of a successful NACT (46). The association between proliferative activity and perfusion has been shown in cell and *ex vivo* studies as central to tumour development (47, 48). Ki-67 was positively correlated with median (35) and mean (49) tumour perfusion fraction respectively in cross-sectional studies. Thus, an increase in proliferative activity has a corresponding increase in volume fraction between capillary blood and tissue water. Bayesian-derived perfusion fraction showed that good responders with a greater decrease in Ki-67 across NACT also had a greater decrease in perfusion fraction at one cycle, therefore enhancing the critical evidence in the clinical population from a longitudinal study. However, simultaneous full fitting and segmented analyses showed no correlation between change in perfusion fraction and Ki-67. A decrease from high pre-NACT (>35%) to low post-NACT (<15%) Ki-67 showed a sustained low recurrence (<20%) at 3 years after diagnosis (32), and post-NACT Ki-67 proliferative index is an independent prognostic marker in addition to pCR (32). The results showed the potential of perfusion fraction in early response for treatment-altered prognosis and the clinical relevance of an imaging biomarker in the targeted evaluation of the impact of NACT on breast tumours.

There was no significant difference in alteration of tissue diffusion *D* between responder groups, indicating that cellularity might not be the correct biological target to reveal the effectiveness of NACT at Cycle 1. It has been shown that an increase in tissue diffusion at the second (34, 41) and third (46) cycle was associated with good response in NACT; however, the time points are at a later stage of NACT and metabolic change at an earlier time point might precede morphological change in cellularity and hence tissue diffusion (34, 41, 46). There was a limited number of cytological or histological studies on changes in cellularity and metabolism during the early phase of NACT, potentially due to the heterogeneity across tumour and the fact that biopsy suffers from partial sampling error. There was a decrease in cellularity in biopsy obtained from good responders after two cycles of NACT (50), although the authors in the current study did not find any study on the direct assessment of cellularity after one cycle of conventional NACT. However, a low tumour cellularity in biopsy at day 15 in patients treated with anti-HER-based chemotherapy (including lapatinib and trastuzumab) (51) and a decrease in cellular proliferative activity of Ki-67 after one cycle of conventional NACT (52) predicted good responders. There was no significant difference in alteration of pseudodiffusion D* between responder groups, in agreement with previous breast cancer treatment studies (34, 53). The results might be due to the higher variability in vascular blood flow motion within the capillary bed (54). The lack of association between alterations in tissue diffusion and pseudodiffusion against alterations in Ki-67 showed an absence of evidence for a direct link between early response in cellularity and vascular blood flow motion against change in proliferative activity in the course of NACT.

Bayesian algorithm offers a robust assessment, and an improved estimation of perfusion fraction in association with pathology. The results of the study suggest that perfusion fraction might be a sensitive biomarker of NACT to improve treatment planning, reduce side effects, and expedite precision medicine. Mammography and breast ultrasound have been proposed at the halfway point of NACT to measure the residual tumour size using the RECIST criteria (55); however, tumour regression is not an accurate predictor of response at the first (56) or second (34) cycle of NACT. There was no correlation in size reduction with tumour grade decrease after two cycles of NACT (50), and a reduction in size of the tumours was seen in both small and large tumours (57), potentially due to the formation of islands of nonviable tumour cells subsequent to NACT (50). Perfusion fraction has the potential for tumour perfusion rate characterisation and responder identification after the first cycle, and the correlation with the change in Ki-67 showed that perfusion fraction might have a unique prognostic value in response-guided NACT prior to surgical intervention.

This investigation was a prospective, registered clinical trial that recruited consecutive patients, and set timing for individual MRI scans ensured comparability between patients (46). This study on patient data provided important clinical evidence to a previous study that used simulated and volunteer data (38) and showed that the Bayesian model might ensure greater accuracy of perfusion fraction in association with pathology for differentiation between good and poor responders. A threshold might not be clear cut, and hence, IVIM will contribute to NACT early responder identification but not as a standalone test. Future large cohort studies that will give an accurate estimation of sensitivity and specificity are required to demonstrate the potential of the Bayesian IVIM model to support early response markers in breast cancer management. A three-direction acquisition scheme was utilised due to limited acquisition time (58) and potential risk of overfitting with DTI parameters very sensitive to noise (39); however, a six-direction scheme (or more) might be used to mitigate the impact of anisotropy in the breast (39, 59, 60) in a future study. The current analysis might also benefit from a multi-compartmental IVIM model to account for the exchange between the extracellular and intracellular compartment that affects the quantification of diffusion and pseudodiffusion, since there was a characteristic change in cellular fibrous tissue (stroma) after NACT and the stromal component of the tumour is critical in tumour biology (50, 61).

## 5 Conclusion

The alteration in perfusion fraction from the Bayesian IVIM model supported the differentiation of good responders from poor responders at the first treatment cycle, and warrants further investigation in comparison to full fitting and segmented analyses in large cohort studies. Early treatment-induced changes in perfusion fraction might serve as non-invasive biomarker to facilitate the delivery of response-guided NACT and the development of an optimal treatment plan.

## Data availability statement

The raw data supporting the conclusions of this article will be made available by the authors, without undue reservation.

## Ethics statement

The studies involving humans were approved by London Research Ethics Committee. The studies were conducted in accordance with the local legislation and institutional requirements. The participants provided their written informed consent to participate in this study.

## Author contributions

SMC: Data curation, Formal Analysis, Investigation, Methodology, Project administration, Validation, Visualization, Writing – original draft, Writing – review & editing. W-SW: Formal Analysis, Investigation, Methodology, Writing – original draft. NS: Data curation, Investigation, Project administration, Writing – review & editing. RS: Conceptualization, Funding acquisition, Investigation, Supervision, Writing – review & editing. TM: Conceptualization, Funding acquisition, Investigation, Supervision, Writing – review & editing. TG: Conceptualization, Funding acquisition, Investigation, Supervision, Writing – review & editing. EH: Conceptualization, Formal Analysis, Funding acquisition, Investigation, Supervision, Writing – review & editing. YM: Conceptualization, Funding acquisition, Investigation, Supervision, Writing – review & editing. JH: Conceptualization, Funding acquisition, Investigation, Project administration, Resources, Supervision, Writing – original draft, Writing – review & editing.

## Footnotes

1 Dickie, B.R., Ahmed, Z., Arvidsson, J., Bell, L.C., Buckley, D.L., Debus, C., et al. (2023). A community-endorsed open-source lexicon for contrast agent-based perfusion MRI: A consensus guidelines report from the ISMRM Open Science Initiative for Perfusion Imaging (OSIPI). Magn Reson Med. Online ahead of print. doi: 10.1002/mrm.29840
