# Supplementary material for: Towards detection of early response in neoadjuvant chemotherapy of breast cancer using Bayesian intravoxel incoherent motion
Source: Front Oncol. 2023 Dec 6;13:1277556. doi: 10.3389/fonc.2023.1277556 (PMC10731248; doi:10.3389/fonc.2023.1277556)
Supplement: Supplementary file 1 [file DataSheet_1.pdf]

Supplementary Material

Appendix A: Supplementary Methods, Tables and Figures for the clinical study

1 Supplementary Methods

Seventeen patients (age 37 – 71 years) with invasive ductal carcinoma of the breast participated in the clinical trial. The baseline and Cycle 1 intravoxel incoherent motion (IVIM) MRI scans were acquired from all participants for analysis.

IVIM Algorithms

Bayesian probability (BP) algorithm, along with three algorithms commonly used in solving IVIM model, including nonlinear least squares (Free), segmented-unconstrained (SU) and segmented-constrained (SC) were implemented (1). All four algorithms used a set of initial values, upper and lower constraints in accordance with a literature on breast (2). The initial values were $f = 0.3$, $D = 0.001$ mm^2/s and $D* = 0.003$ mm^2/s. The upper bounds were $f = 0.5$, assuming pseudodiffusion was not dominating in voxel, $D = 0.003$ mm^2/s and $D* = 0.3$ mm^2/s. The lower bounds were $f = 0$, $D = 0$ mm^2/s and $D* = 0$ mm^2/s.

Nonlinear least squares (Free): The IVIM-derived parameters were estimated using the bi-exponential equation (3):

$$\frac{S_b}{S_0} = (1-f)e^{-bD} + fe^{-bD*} \qquad \text{(A.1)}$$

where $\frac{S_b}{S_0}$ is the overall signal attenuation, f is the fraction of compartment attributed to capillary blood flow against tissue water, D is the molecular diffusion coefficient attributed to the mean diffusivity of the tissue and $D*$ is the pseudodiffusion coefficient attributed to the vascular blood flow motion. The curve fitting was based on trust region algorithm, allowing the implementation of constraints to

perform a simultaneous fit of *f*, *D* and $D*$.

Segmented-unconstrained (SU): The SU model assumed that when $D*$ >> *D*, the impact of $D*$ was negligible above a high *b*-value (> 200 s/mm^2) (4). The equation can be rewritten as

$$S_b = S_o' e^{-bD} \qquad (A.2)$$

First, the data acquired with *b*-values from 250 s/mm^2 to 1000 s/mm^2 was fitted with equation (A.2) to estimate *D* and zero-intercept S_o'. The perfusion fraction *f* was then calculated by

$$f = 1 - \frac{S_0'}{S_o} \qquad (A.3)$$

where S_o was the measured signal at $b = 0$ s/mm^2. Last, the data acquired at all *b*-values was fitted with equation (A.1), with $D*$ as the only free parameter, using nonlinear least square method based on trust region algorithm.

Segmented-constrained (SC): The initial approach of SC model was identical to SU, and estimated *D* based on mono-exponential equation (A.2) with *b*-values higher than 200 s/mm^2. Then, *f* and $D*$ were estimated by nonlinear least square fitting of equation (A.1) based on trust region algorithm (4).

<u>Image Analysis</u>

The data analysis and curve fitting algorithms were performed in MATLAB (R2020a, Mathworks, Natrick, MA, USA). Data was processed on a voxel-by-voxel basis, and all lesions were identified on dynamic contrast enhanced MRI and IVIM images acquired at $b = 1000$ s/mm^2. The tumour region-of-interests (ROIs) were delineated manually in ImageJ (v1.58k, National Institute of Health, Bethesda, MD, USA), with boundary restricted inside the tumour edge to avoid partial volume artefact. The diffusion and pseudodiffusion signals within ROIs at Baseline and Cycle 1 were analysed. Further details are given in the Methods section of the main text.

Statistical Analysis

Statistical analysis was performed in *R* (v3.6.3, The *R* Foundation for Statistical Computing, Vienna, Austria). The normality of the distribution was assessed using Shapiro-Wilk test. The difference in baseline and percentage change in perfusion fraction, diffusion and pseudodiffusion between good and poor responders was compared using Wilcoxon rank sum test. The correlation of baseline and percentage change in perfusion fraction, diffusion and pseudodiffusion against Ki-67 in core biopsy and percentage change in Ki-67 respectively was performed using Spearman's rank correlation test. A p value < 0.05 was considered statistically significant.

2 Supplementary Tables

Table A1. Comparison of IVIM-derived parameters between responder groups before and after first cycle of NACT and the association with Ki-67

The baseline and percentage change in perfusion fraction (*f*), tissue diffusion (*D*) and pseudodiffusion (*D**) in good responders and poor responders from nonlinear least squares (Free), segmented-unconstrained (SU) and segmented-constrained (SC) analyses. Values are presented as median (IQR).

IVIM-derived parameters	Baseline-*f/D/D**			%Change-*f/D/D**			Ki-67 correlations (ρ score, *p* value)	
	All (n=17)	Good Responder (n=8)	Poor Responder (n=9)	All (n=16)	Good Responder (n=7)[a]	Poor Responder (n=9)	Core[b]	%Change[c]
Free								
f[d]	22.43 (18.53 – 28.66)	21.54 (16.90 – 26.46)	23.49 (18.68 – 30.54)	-1.38 (-11.13 – 13.74)	-2.50 (-24.92 – 3.59)	1.96 (-3.99 – 19.97)	0.030, 0.918	0.380, 0.217
D	1.39 (1.04 – 1.74)	1.39 (1.03 – 1.94)	1.39 (1.04 – 1.43)	16.77 (5.97 – 46.02)	42.22 (2.32 – 82.63)	11.00 (8.97 – 25.04)	-0.150, 0.567	0.380, 0.226
*D**	4.80 (2.34 – 6.71)	3.81 (2.29 – 5.08)	5.01 (3.99 – 7.05)	-17.71 (-52.06 – 59.32)	25.16 (-33.54 – 51.62)	-20.20 (-70.79 – 58.35)	-0.240, 0.348	0.240, 0.443
SU								
f[d]	11.18 (9.69 – 12.86)	10.25 (9.08 – 13.14)	11.20 (10.22 – 11.85)	-1.10 (-14.49 – 10.78)	-13.74 (-28.08 – -5.21)	3.84 (1.14 – 14.99)	0.120, 0.653	0.380, 0.217
D	0.99 (0.92 – 1.29)	1.01 (0.86 – 1.56)	0.99 (0.92 – 1.23)	16.10 (1.39 – 40.70)	28.85 (7.97 – 58.64)	13.99 (0.86 – 26.89)	-0.270, 0.291	0.210, 0.513
*D**	11.72 (10.43 – 15.84)	11.86 (10.05 – 16.70)	11.72 (10.58 – 13.56)	16.88 (-0.09 – 40.39)	30.92 (-4.28 – 52.83)	15.32 (5.41 – 31.40)	-0.220, 0.390	0.380, 0.226
SC								
f[d]	10.88 (9.71 – 11.74)	10.26 (9.23 – 11.35)	11.28 (9.99 – 11.74)	4.94 (-5.11 – 15.01)	-1.28 (-15.50 – 3.44)	8.68 (3.50 – 31.97)	0.170, 0.504	0.340, 0.286
D	0.99 (0.92 – 1.29)	1.01 (0.86 – 1.56)	0.99 (0.92 – 1.23)	16.10 (1.39 – 40.70)	28.85 (7.97 – 58.64)	13.99 (0.86 – 26.89)	-0.270, 0.291	0.210, 0.513
*D**	9.91 (8.39 – 11.93)	9.41 (8.62 – 10.62)	10.58 (8.39 – 11.94)	-8.62 (-13.29 – 1.11)	-11.69 (-22.47 – -2.71)	-8.28 (-9.34 – 6.09)	0.100, 0.694	0.430, 0.208

[a] One patient did not complete MR scan due to complications.
[b] Spearman's rank correlation test – baseline-*f/D/D** vs Ki-67 Core.
[c] Spearman's rank correlation test – %Change-*f/D/D** vs %Change-Ki-67.
[d] Units at baseline – *f*: percentage (%), *D* and *D**: $\times 10^{-3}$ mm^2/s.

3 Supplementary Figures

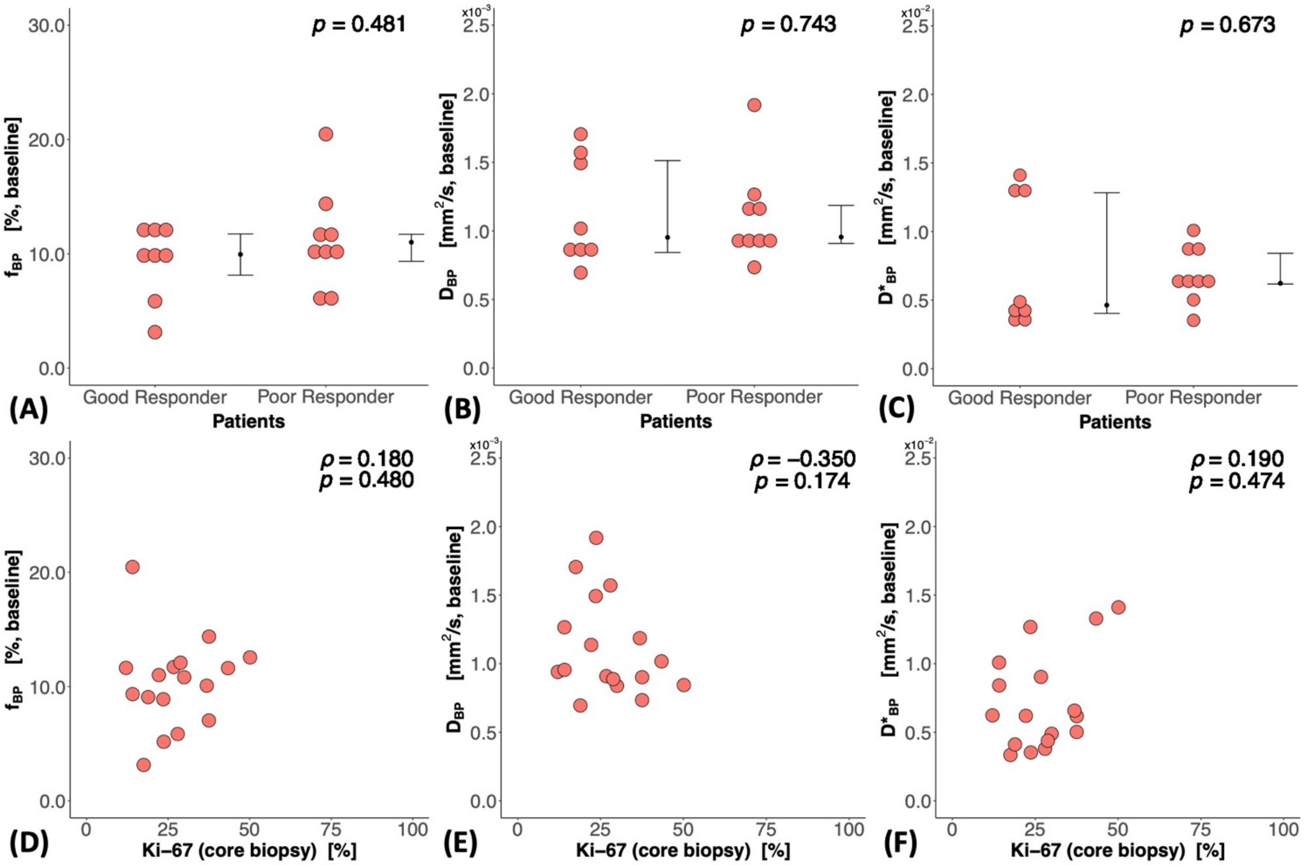

Figure A1. Baseline perfusion fraction (*f*), tissue diffusion (*D*) and pseudodiffusion (*D) between good and poor responders from Bayesian probability (BP) IVIM model and correlation with Ki-67 in core biopsy**

The baseline (A) *f*, (B) *D* and (C) *D** between good and poor responders from BP algorithm are shown in dot plots. The correlation of (D) *f*, (E) *D* and (F) *D** at Baseline from BP algorithm against tumour cellular proliferation marker Ki-67 in core biopsy are shown in scatter plots. Each dot represents the IVIM-derived parameter of an individual patient. Error bar represents median (IQR). Spearman's rank correlation coefficient (*rho* (ρ)) was used for correlation analysis and respective ρ score and *p*-value are shown on each plot.

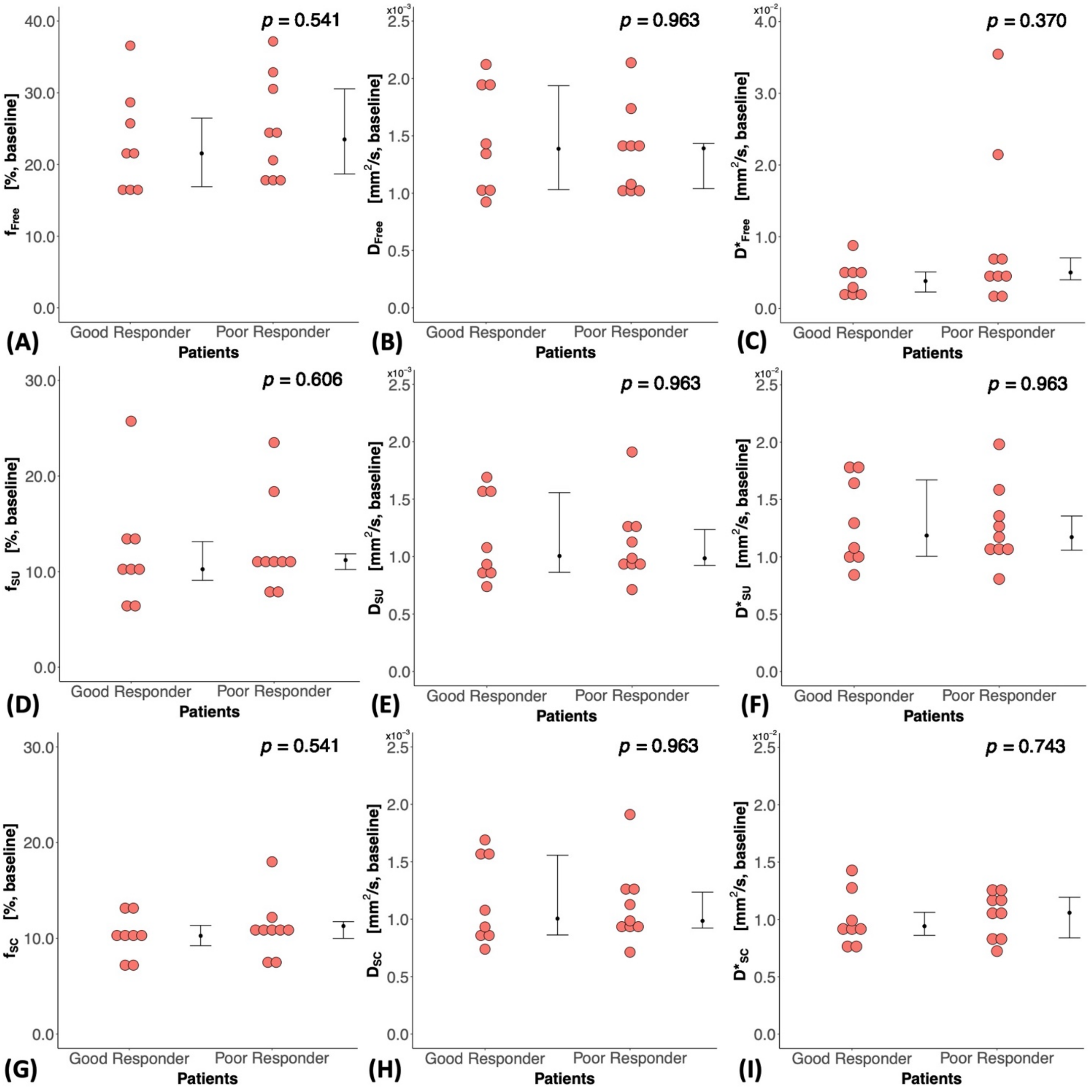

Figure A2. Baseline perfusion fraction (*f*), tissue diffusion (*D*) and pseudodiffusion (*D) between good and poor responders from nonlinear least squares (Free), segmented-unconstrained (SU) and segmented-constrained (SC) IVIM models**

The baseline *f*, *D* and *D** between good and poor responders from (A-C) Free, (D-F) SU and (G-I) SC algorithms are shown in dot plots. Each dot represents the IVIM-derived parameter of an individual patient. Error bar represents median (IQR). Statistical *p* values are shown on the upper right corner.

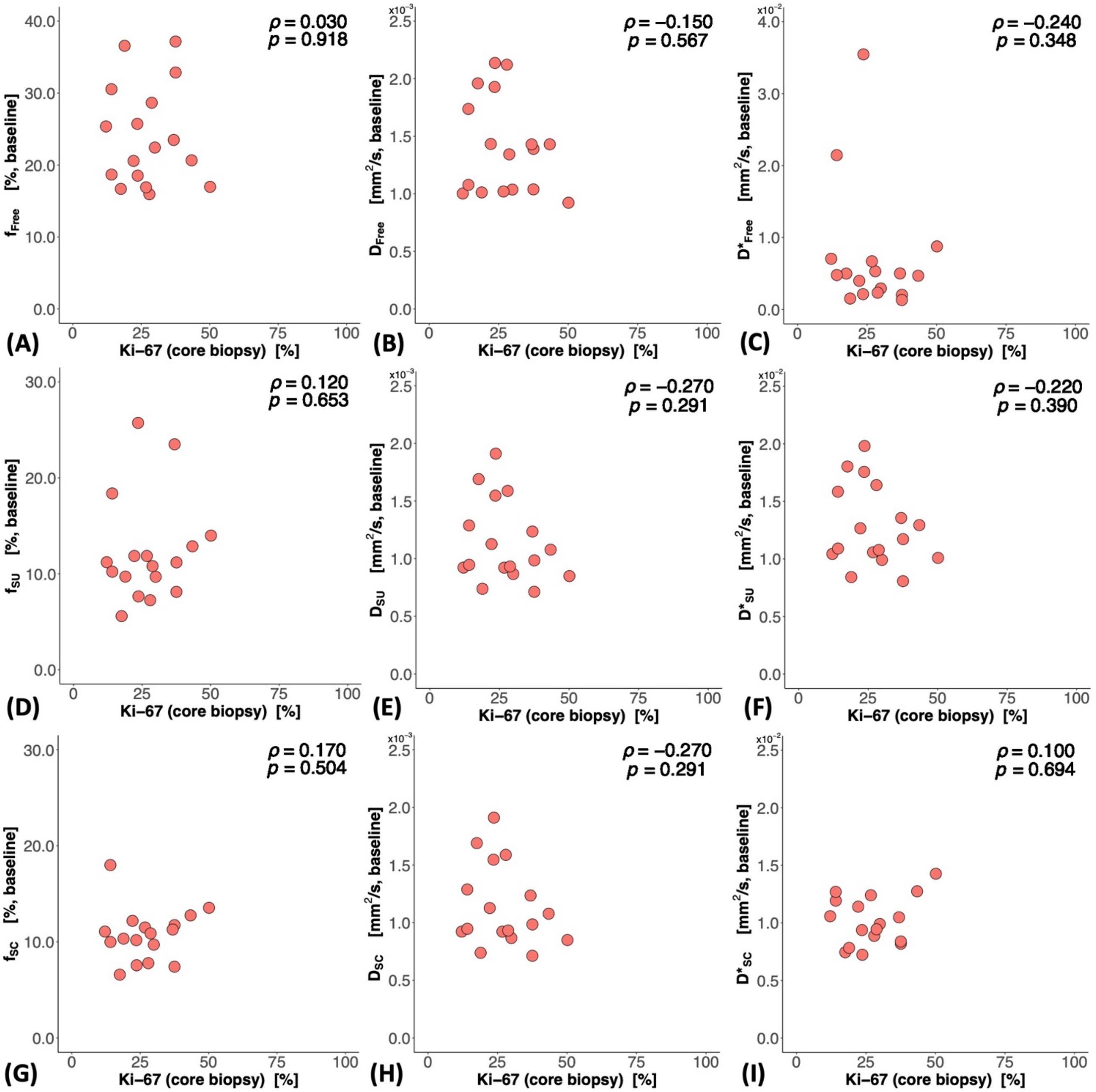

Figure A3. Baseline perfusion fraction (*f*), tissue diffusion (*D*) and pseudodiffusion (*D) against Ki-67 in core biopsy**

The correlations of *f*, *D* and *D** from (A-C) Nonlinear least squares (Free), (D-F) Segmented-unconstrained (SU) and (G-I) Segmented-constrained (SC) algorithms at Baseline against tumour cellular proliferation marker Ki-67 in core biopsy are shown in scatter plots. Spearman's rank correlation coefficient (*rho* (ρ)) was used for correlation analysis and respective ρ score and *p*-value are shown on each plot.

4 References

1. Barbieri S, Donati OF, Froehlich JM, Thoeny HC. Impact of the calculation algorithm on biexponential fitting of diffusion-weighted MRI in upper abdominal organs. Magn Reson Med. 2016;75(5):2175-84.

2. Vidić I, Jerome NP, Bathen TF, Goa PE, While PT. Accuracy of breast cancer lesion classification using intravoxel incoherent motion diffusion-weighted imaging is improved by the inclusion of global or local prior knowledge with bayesian methods. J Magn Reson Imaging. 2019;50(5):1478-88.

3. Liu C, Liang C, Liu Z, Zhang S, Huang B. Intravoxel incoherent motion (IVIM) in evaluation of breast lesions: comparison with conventional DWI. Eur J Radiol. 2013;82(12):e782-9.

4. Bedair R, Priest AN, Patterson AJ, McLean MA, Graves MJ, Manavaki R, et al. Assessment of early treatment response to neoadjuvant chemotherapy in breast cancer using non-mono-exponential diffusion models: a feasibility study comparing the baseline and mid-treatment MRI examinations. Eur Radiol. 2017;27(7):2726-36.
